# The genome sequence of a basidiomycete yeast, *Tausonia pullulans *(Lindner) X.Z. Liu, F.Y. Bai, M. Groenew. & Boekhout, 2016 (Mrakiaceae)

**DOI:** 10.12688/wellcomeopenres.23350.1

**Published:** 2024-11-20

**Authors:** Richard Wright, Brian Douglas, Royal Botanic Gardens Kew Genome Acquisition Lab, Darwin Tree of Life Barcoding collective, Wellcome Sanger Institute Tree of Life Management, Samples and Laboratory team, Wellcome Sanger Institute Scientific Operations: Sequencing Operations, Wellcome Sanger Institute Tree of Life Core Informatics team, Tree of Life Core Informatics collective, Darwin Tree of Life Consortium

**Affiliations:** 1 Cardiff University, Cardiff, Wales, UK; 2 Royal Botanic Gardens Kew, Richmond, England, UK

**Keywords:** Tausonia pullulans, basidiomycete yeast, genome sequence, chromosomal, Cystofilobasidiales

## Abstract

We present a genome assembly from an individual *Tausonia pullulans* (a basidiomycete yeast; Basidiomycota; Tremellomycetes; Cystofilobasidiales; Mrakiaceae). The genome sequence is 23.9 megabases in span. Most of the assembly is scaffolded into 20 chromosomal pseudomolecules. The mitochondrial genome has also been assembled and is 18.82 kilobases in length.

## Species taxonomy

Eukaryota; Opisthokonta; Fungi; Dikarya; Basidiomycota; Tremellomycetes; Tremellomycetidae; Cystofilobasidiales; Mrakiaceae; *Tausonia*; *Tausonia pullulans* ((Lindner) Xin Zhan Liu, F.Y. Bai, M. Groenew. & Boekhout, 2015) (NCBI:txid82525)

## Background

*Tausonia pullulans* is a psychrotolerant basidiomycete yeast in Tremellomycetes ( Trochine *et al.*, 2022a). It produces budding cells that are ovoid to cylindrical and may be enteroblastic. It can produce extensive true hyphae that disarticulate to form arthroconidia, its key method of asexual reproduction ( Fell & Guého-Kellermann, 2011; Fell & Scorzetti, 2004). It is not known to sexually reproduce and lacks the clamp connections and chlamydospore production of the closely related *T. pamirica* ( Sampaio, 2011).

This yeast is known from all continents ( GBIF, 2023), from a broad range of substrates and habitats, including records from soil, herbaceous plants, trees, pigeon faeces, sawdust, tree sap of birch, maple, and chestnut, butter, frozen beef, packaged kimchi ( Kim *et al.*, 2020) and a nasal infection in a cat ( Fell & Guého-Kellermann, 2011). *T. pullulans* is most frequently reported from soils ( Yurkov, 2018) and woody substrates ( Cadete *et al.*, 2017).

It is known to produce higher levels of lignocellulose decay enzymes, as well as glucoamylases and pectinolytic enzymes, when compared to other Tremellomycetes ( Trochine *et al.*, 2022a), and has shown cellulolytic activity ( Dennis, 1972).

This species has gone through a number of taxonomic rearrangements, first described as *Oidium pullulans* by Lindner in 1901, sampled from storage barrels in an experimental brewery ( Lindner, 1901). Most recently it was transferred from *Trichosporon* to a new monotypic genus, *Guehomyces* ( Fell & Scorzetti, 2004), based on DNA; and subsequently moved to *Tausonia* as part of a phylogenetic reclassification of the Tremellomycetes based on analyses of seven gene regions ( Liu *et al.*, 2015).

*T. pullulans*’ enzymatic activities have been applied to the degradation of industrial pollutants ( Romero *et al.*, 2002), wood pulping processes ( Sláviková *et al.*, 2002), hydrolysis of lactose in milk ( Nakagawa *et al.*, 2006), pectin extraction from limes ( Bezus *et al.*, 2022), prevention of post-harvest decay in pears ( Yao *et al.*, 2004), and starch hydrolysis ( Trochine *et al.*, 2022b), amongst other applications.

Although reported as human pathogen by Moylett *et al.* (2003), it is considered unlikely due to it being a low temperature organism which has a maximum growth temperature of 25 °C ( Fell & Guého-Kellermann, 2011).

*T. pullulans* represents an ecologically interesting species that may play an important role in the wood decay process and has many potential biotechnology applications. Further discoveries, alongside improved taxonomic understanding, will be aided by the production of this genome.

## Genome sequence report

The genome was sequenced from a specimen of *Tausonia pullulans* from Siston, Gloucestershire, UK. A total of 458-fold coverage in Pacific Biosciences single-molecule HiFi long reads was generated. Primary assembly contigs were scaffolded with chromosome conformation Hi-C data. Manual assembly curation corrected 12 missing joins or mis-joins, reducing the scaffold number by 12.00%, and decreasing the scaffold N50 by 5.78%.

**Figure 1.**
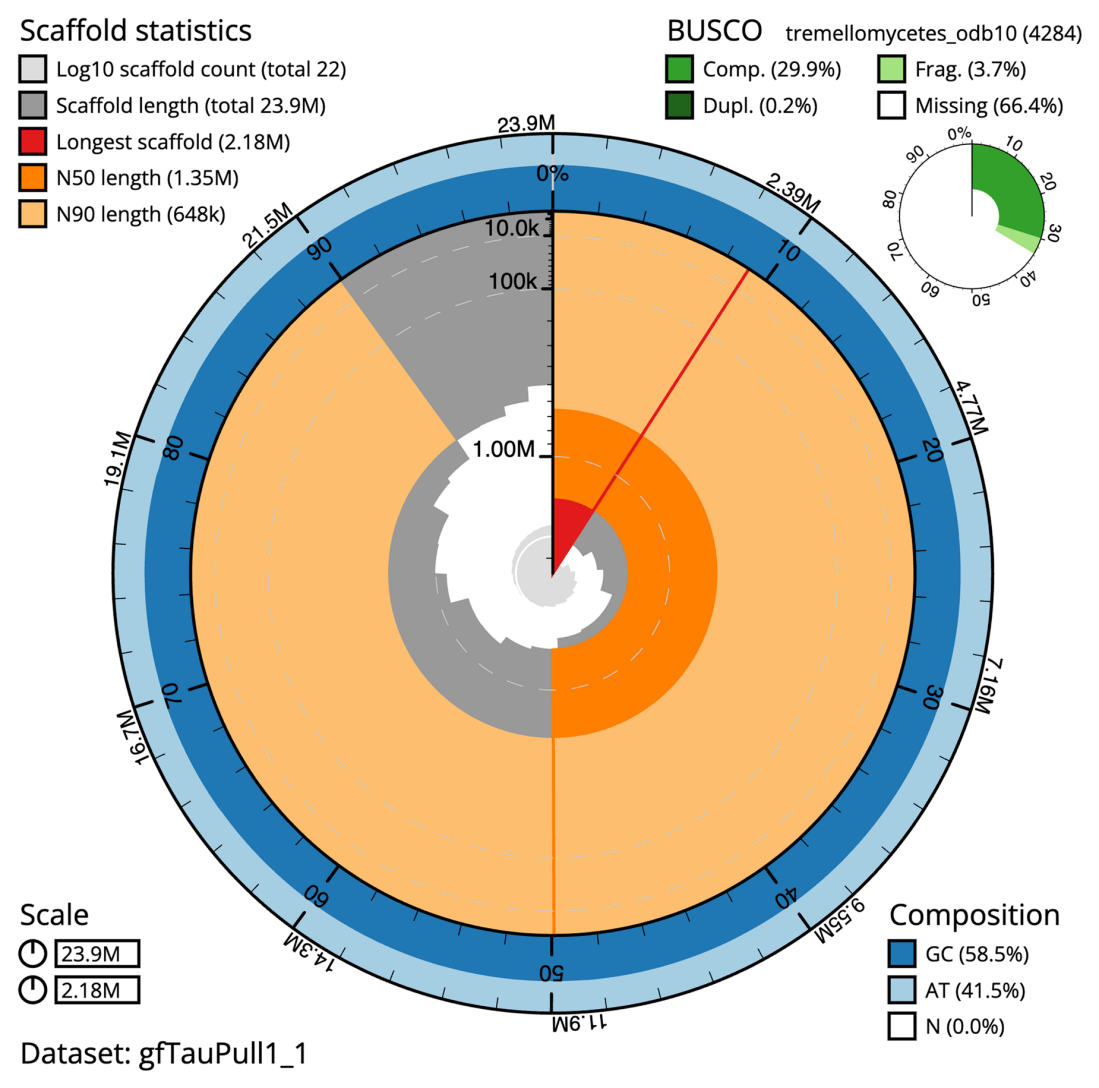
Genome assembly of *Tausonia pullulans*, gfTauPull1.1: metrics. The BlobToolKit Snailplot shows N50 metrics and BUSCO gene completeness. The main plot is divided into 1,000 bins around the circumference with each bin representing 0.1% of the 23,873,987 bp assembly. The distribution of scaffold lengths is shown in dark grey with the plot radius scaled to the longest scaffold present in the assembly (2,178,134 bp, shown in red). Orange and pale-orange arcs show the N50 and N90 scaffold lengths (1,354,748 and 647,864 bp), respectively. The pale grey spiral shows the cumulative scaffold count on a log scale with white scale lines showing successive orders of magnitude. The blue and pale-blue area around the outside of the plot shows the distribution of GC, AT and N percentages in the same bins as the inner plot. A summary of complete, fragmented, duplicated and missing BUSCO genes in the tremellomycetes_odb10 set is shown in the top right. An interactive version of this figure is available at https://blobtoolkit.genomehubs.org/view/gfTauPull1_1/dataset/gfTauPull1_1/snail.

**Figure 2.**
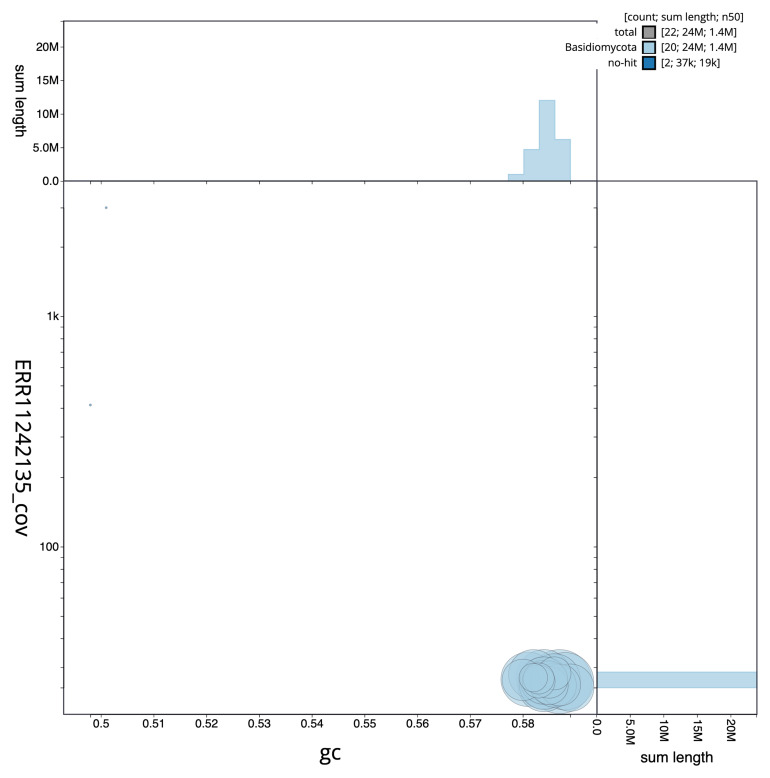
Genome assembly of *Tausonia pullulans*, gfTauPull1.1: BlobToolKit GC-coverage plot. Scaffolds are coloured by phylum. Circles are sized in proportion to scaffold length. Histograms show the distribution of scaffold length sum along each axis. An interactive version of this figure is available at https://blobtoolkit.genomehubs.org/view/gfTauPull1_1/dataset/gfTauPull1_1/blob.

**Figure 3.**
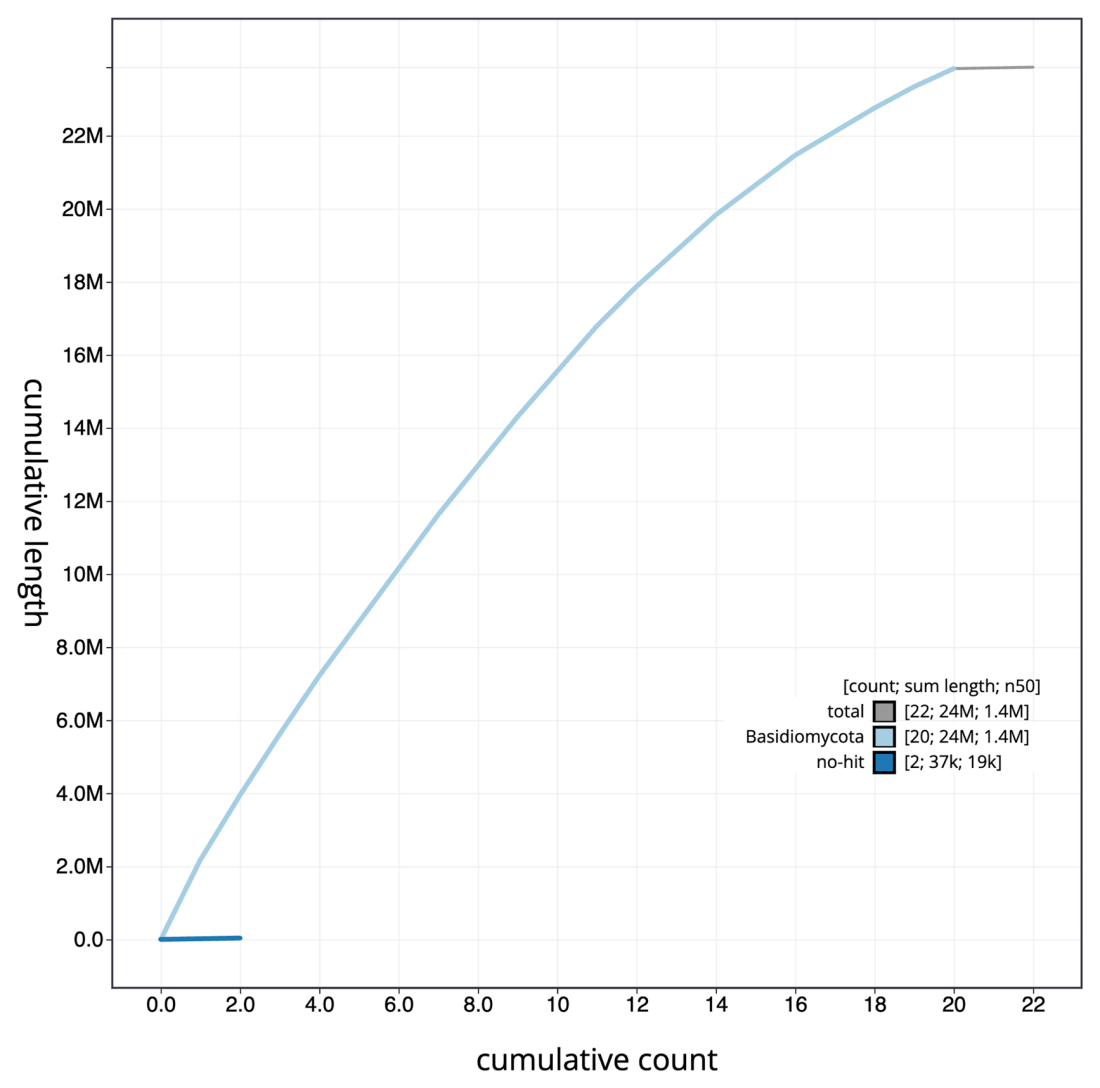
Genome assembly of *Tausonia pullulans*, gfTauPull1.1: BlobToolKit cumulative sequence plot. The grey line shows cumulative length for all scaffolds. Coloured lines show cumulative lengths of scaffolds assigned to each phylum using the buscogenes taxrule. An interactive version of this figure is available at https://blobtoolkit.genomehubs.org/view/gfTauPull1_1/dataset/gfTauPull1_1/cumulative.

**Figure 4.**
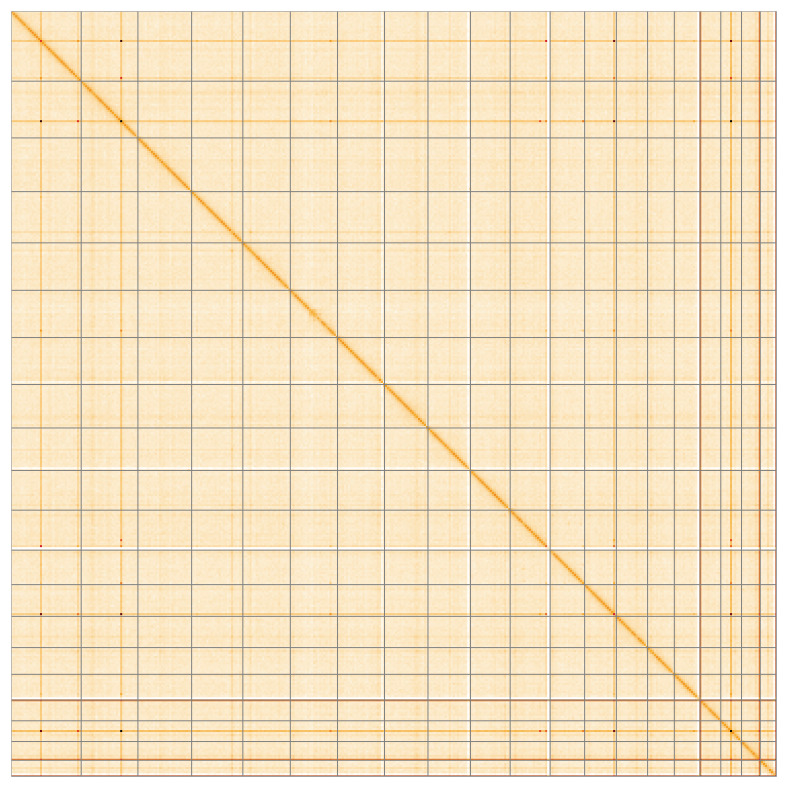
Genome assembly of *Tausonia pullulans*, gfTauPull1.1: Hi-C contact map of the gfTauPull1.1 assembly, visualised using HiGlass. Chromosomes are shown in order of size from left to right and top to bottom. An interactive version of this figure may be viewed at https://genome-note-higlass.tol.sanger.ac.uk/l/?d=BU4zDe9QSj6FbzspKlCP2A.

The final assembly has a total length of 23.9 Mb in 21 sequence scaffolds with a scaffold N50 of 1.4 Mb ( Table 1). The snail plot in Figure 1 provides a summary of the assembly statistics, while the distribution of assembly scaffolds on GC proportion and coverage is shown in Figure 2. The cumulative assembly plot in Figure 3 shows curves for subsets of scaffolds assigned to different phyla. Most (99.84%) of the assembly sequence was assigned to 20 chromosomal-level scaffolds, representing 20 autosomes. Chromosome-scale scaffolds confirmed by the Hi-C data are named in order of size ( Figure 4; Table 2). While not fully phased, the assembly deposited is of one haplotype. Contigs corresponding to the second haplotype have also been deposited. The mitochondrial genome was also assembled and can be found as a contig within the multifasta file of the genome submission.

**Table 1.** Genome data for *Tausonia pullulans*, gfTauPull1.1.

Project accession data		
Assembly identifier	gfTauPull1.1	
Species	*Tausonia pullulans*	
Specimen	gfTauPull1	
NCBI taxonomy ID	82525	
BioProject	PRJEB61351	
BioSample ID	SAMEA13759877	
Isolate information	gfTauPull1 (DNA, HiC and RNA)	
Assembly metrics *		*Benchmark*
Consensus quality (QV)	71.1	*≥ 50*
*k*-mer completeness	100.0%	*≥ 95%*
BUSCO **	C:29.9%[S:29.7%,D:0.2%],F:3.7%,M:66.4%,n:4,284	*C ≥ 95%*
Percentage of assembly mapped to chromosomes	99.84%	*≥ 95%*
Sex chromosomes	None	*localised homologous pairs*
Organelles	Mitochondrial genome: 18.82 kb	*complete single alleles*
Raw data accessions		
PacificBiosciences SEQUEL II	ERR11242134, ERR11242135	
Hi-C Illumina	ERR11242558	
PolyA RNA-Seq Illumina	ERR11242559	
Genome assembly		
Assembly accession	GCA_951802195.1	
*Accession of alternate haplotype*	GCA_951802215.1	
Span (Mb)	23.9	
Number of contigs	58	
Contig N50 length (Mb)	0.7	
Number of scaffolds	21	
Scaffold N50 length (Mb)	1.4	
Longest scaffold (Mb)	2.18	

* Assembly metric benchmarks are adapted from column VGP-2020 of “Table 1: Proposed standards and metrics for defining genome assembly quality” from ( Rhie *et al.*, 2021). ** BUSCO scores based on the tremellomycetes_odb10 BUSCO set using version 5.3.2. C = complete [S = single copy, D = duplicated], F = fragmented, M = missing, n = number of orthologues in comparison. A full set of BUSCO scores is available at https://blobtoolkit.genomehubs.org/view/gfTauPull1_1/dataset/gfTauPull1_1/busco.

**Table 2.** Chromosomal pseudomolecules in the genome assembly of *Tausonia pullulans*, gfTauPull1.

INSDC accession	Chromosome	Length (Mb)	GC%
OX637620.1	1	2.18	58.5
OX637621.1	2	1.77	59.0
OX637622.1	3	1.67	58.5
OX637623.1	4	1.6	58.0
OX637624.1	5	1.48	58.5
OX637625.1	6	1.47	58.5
OX637626.1	7	1.46	58.5
OX637627.1	8	1.35	58.0
OX637628.1	9	1.33	58.5
OX637629.1	10	1.24	58.5
OX637630.1	11	1.24	59.0
OX637631.1	12	1.08	58.5
OX637632.1	13	0.98	58.5
OX637633.1	14	0.97	58.0
OX637634.1	15	0.83	58.5
OX637635.1	16	0.8	58.5
OX637636.1	17	0.65	58.5
OX637637.1	18	0.64	58.5
OX637638.1	19	0.58	58.5
OX637639.1	20	0.5	58.0
OX637640.1	MT	0.02	50.0

The estimated Quality Value (QV) of the final assembly is 71.1 with *k*-mer completeness of 100.0%, and the assembly has a BUSCO v5.3.2 completeness of 29.9% (single = 29.7%, duplicated = 0.2%), using the tremellomycetes_odb10 reference set ( *n* = 4,284).

Metadata for specimens, barcode results, spectra estimates, sequencing runs, contaminants and pre-curation assembly statistics are given at https://links.tol.sanger.ac.uk/species/82525.

## Methods

### Sample acquisition and nucleic acid extraction

The genome was sequenced from a pure culture (SB1_H10a) obtained by Richard Wright from the heartwood of an oak core sample, collected from a standing oak tree at Siston Brook, Bristol, UK (latitude 51.47, longitude –2.45).

A specimen of *Tausonia pullulans* (specimen ID KDTOL00172, ToLID gfTauPull1) was collected from Siston Brook, Siston, Gloucestershire, UK (latitude 51.47, longitude –2.45) on 2021-06-18. The specimen was handpicked and cultured before being transferred to tubes. The specimen was collected by Richard Wright (Royal Botanic Gardens Kew) and identified by Richard Wright and Brian Douglas (Royal Botanic Gardens Kew) and preserved by snap-freezing.

Protocols developed by the Wellcome Sanger Institute (WSI) Tree of Life core laboratory have been deposited on protocols.io ( Denton *et al.*, 2023). The workflow for high molecular weight (HMW) DNA extraction at the WSI includes a sequence of core procedures: sample preparation; sample homogenisation, DNA extraction, fragmentation, and clean-up. In sample preparation, the gfTauPull1 sample was weighed and dissected on dry ice ( Jay *et al.*, 2023). For sample homogenisation, tissue was cryogenically disrupted using the Covaris cryoPREP ^®^ Automated Dry Pulverizer ( Narváez-Gómez *et al.*, 2023).

HMW DNA was extracted using the Automated Plant MagAttract v2 protocol ( Todorovic *et al.*, 2023a). HMW DNA was sheared into an average fragment size of 12–20 kb in a Megaruptor 3 system ( Todorovic *et al.*, 2023b). Sheared DNA was purified by solid-phase reversible immobilisation, using AMPure PB beads to eliminate shorter fragments and concentrate the DNA ( Strickland *et al.*, 2023). The concentration of the sheared and purified DNA was assessed using a Nanodrop spectrophotometer and Qubit Fluorometer and Qubit dsDNA High Sensitivity Assay kit. Fragment size distribution was evaluated by running the sample on the FemtoPulse system.

RNA was extracted from gfTauPull1 in the Tree of Life Laboratory at the WSI using the RNA Extraction: Automated MagMax™ *mir*Vana protocol ( do Amaral *et al.*, 2023). The RNA concentration was assessed using a Nanodrop spectrophotometer and a Qubit Fluorometer using the Qubit RNA Broad-Range Assay kit. Analysis of the integrity of the RNA was done using the Agilent RNA 6000 Pico Kit and Eukaryotic Total RNA assay.

### Sequencing

Pacific Biosciences HiFi circular consensus DNA sequencing libraries were constructed according to the manufacturers’ instructions. Poly(A) RNA-Seq libraries were constructed using the NEB Ultra II RNA Library Prep kit. DNA and RNA sequencing was performed by the Scientific Operations core at the WSI on Pacific Biosciences SEQUEL II (HiFi) and Illumina NovaSeq 6000 (RNA-Seq) instruments. Hi-C data were also generated from cells of gfTauPull1 using the Arima2 kit and sequenced on the Illumina NovaSeq 6000 instrument.

### Genome assembly, curation and evaluation

Assembly was carried out with Hifiasm ( Cheng *et al.*, 2021) and haplotypic duplication was identified and removed with purge_dups ( Guan *et al.*, 2020). The assembly was then scaffolded with Hi-C data ( Rao *et al.*, 2014) using YaHS ( Zhou *et al.*, 2023). The assembly was checked for contamination and corrected as described previously ( Howe *et al.*, 2021). Manual curation was performed using HiGlass ( Kerpedjiev *et al.*, 2018) and PretextView ( Harry, 2022). The mitochondrial genome was assembled using MitoHiFi ( Uliano-Silva *et al.*, 2023), which runs MitoFinder ( Allio *et al.*, 2020) and uses these annotations to select the final mitochondrial contig and to ensure the general quality of the sequence.

A Hi-C map for the final assembly was produced using bwa-mem2 ( Vasimuddin *et al.*, 2019) in the Cooler file format ( Abdennur & Mirny, 2020). To assess the assembly metrics, the *k*-mer completeness and QV consensus quality values were calculated in Merqury ( Rhie *et al.*, 2020). This work was done using Nextflow ( Di Tommaso *et al.*, 2017) DSL2 pipelines “sanger-tol/readmapping” ( Surana *et al.*, 2023a) and “sanger-tol/genomenote” ( Surana *et al.*, 2023b). The genome was analysed within the BlobToolKit environment ( Challis *et al.*, 2020) and BUSCO scores ( Manni *et al.*, 2021) were calculated.

Table 3 contains a list of relevant software tool versions and sources.

**Table 3.** Software tools: versions and sources.

Software tool	Version	Source
BlobToolKit	4.1.7	https://github.com/blobtoolkit/blobtoolkit
BUSCO	5.3.2	https://gitlab.com/ezlab/busco
Hifiasm	0.16.1-r375	https://gitlab.com/chhylp123/hifiasm
HiGlass	1.11.6	https://gitlab.com/higlass/higlass
Merqury	MerquryFK	https://gitlab.com/thegenemyers/MERQURY.FK
MitoHiFi	2	https://gitlab.com/marcelauliano/MitoHiFi
PretextView	0.2	https://gitlab.com/wtsi-hpag/PretextView
purge_dups	1.2.3	https://gitlab.com/dfguan/purge_dups
sanger-tol/genomenote	v1.0	https://gitlab.com/sanger-tol/genomenote
sanger-tol/readmapping	1.1.0	https://gitlab.com/sanger-tol/readmapping/tree/1.1.0
YaHS	yahs-1.1.91eebc2	https://gitlab.com/c-zhou/yahs

### Wellcome Sanger Institute – Legal and Governance

The materials that have contributed to this genome note have been supplied by a Darwin Tree of Life Partner. The submission of materials by a Darwin Tree of Life Partner is subject to the **‘Darwin Tree of Life Project Sampling Code of Practice’**, which can be found in full on the Darwin Tree of Life website here. By agreeing with and signing up to the Sampling Code of Practice, the Darwin Tree of Life Partner agrees they will meet the legal and ethical requirements and standards set out within this document in respect of all samples acquired for, and supplied to, the Darwin Tree of Life Project.

Further, the Wellcome Sanger Institute employs a process whereby due diligence is carried out proportionate to the nature of the materials themselves, and the circumstances under which they have been/are to be collected and provided for use. The purpose of this is to address and mitigate any potential legal and/or ethical implications of receipt and use of the materials as part of the research project, and to ensure that in doing so we align with best practice wherever possible. The overarching areas of consideration are:

•  Ethical review of provenance and sourcing of the material

•  Legality of collection, transfer and use (national and international)

Each transfer of samples is further undertaken according to a Research Collaboration Agreement or Material Transfer Agreement entered into by the Darwin Tree of Life Partner, Genome Research Limited (operating as the Wellcome Sanger Institute), and in some circumstances other Darwin Tree of Life collaborators.

## Data Availability

European Nucleotide Archive: *Tausonia pullulans*. Accession number PRJEB61351; https://identifiers.org/ena.embl/PRJEB61351. The genome sequence is released openly for reuse. The *Tausonia pullulans* genome sequencing initiative is part of the Darwin Tree of Life (DToL) project. All raw sequence data and the assembly have been deposited in INSDC databases. The genome will be annotated using available RNA-Seq data and presented through the Ensembl pipeline at the European Bioinformatics Institute. Raw data and assembly accession identifiers are reported in Table 1.

## Author information

Members of the Royal Botanic Gardens Kew Genome Acquisition Lab are listed here: https://doi.org/10.5281/zenodo.4786680.

Members of the Darwin Tree of Life Barcoding collective are listed here: https://doi.org/10.5281/zenodo.4893703.

Members of the Wellcome Sanger Institute Tree of Life Management, Samples and Laboratory team are listed here: https://doi.org/10.5281/zenodo.10066175.

Members of Wellcome Sanger Institute Scientific Operations: Sequencing Operations are listed here: https://doi.org/10.5281/zenodo.10043364.

Members of the Wellcome Sanger Institute Tree of Life Core Informatics team are listed here: https://doi.org/10.5281/zenodo.10066637.

Members of the Tree of Life Core Informatics collective are listed here: https://doi.org/10.5281/zenodo.5013541.

Members of the Darwin Tree of Life Consortium are listed here: https://doi.org/10.5281/zenodo.4783558.

## References

[ref-1] Abdennur N Mirny LA : Cooler: scalable storage for Hi-C data and other genomically labeled arrays. *Bioinformatics.* 2020;36(1):311–316. 10.1093/bioinformatics/btz540 31290943 PMC8205516

[ref-2] Allio R Schomaker-Bastos A Romiguier J : MitoFinder: efficient automated large-scale extraction of mitogenomic data in target enrichment phylogenomics. *Mol Ecol Resour.* 2020;20(4):892–905. 10.1111/1755-0998.13160 32243090 PMC7497042

[ref-3] Bezus B Esquivel JCC Cavalitto S : Pectin extraction from lime pomace by cold-active polygalacturonase-assisted method. *Int J Biol Macromol.* 2022;209(Pt A):290–298. 10.1016/j.ijbiomac.2022.04.019 35398384

[ref-4] Cadete RM Lopes MR Rosa CA : Yeasts associated with decomposing plant material and rotting wood. *Yeasts in natural ecosystems: diversity.* 2017;265–292. 10.1007/978-3-319-62683-3_9

[ref-5] Challis R Richards E Rajan J : BlobToolKit – interactive quality assessment of genome assemblies. *G3 (Bethesda).* 2020;10(4):1361–1374. 10.1534/g3.119.400908 32071071 PMC7144090

[ref-6] Cheng H Concepcion GT Feng X : Haplotype-resolved *de novo* assembly using phased assembly graphs with hifiasm. *Nat Methods.* 2021;18(2):170–175. 10.1038/s41592-020-01056-5 33526886 PMC7961889

[ref-7] Dennis C : Breakdown of cellulose by Yeast Species. *Microbiology.* 1972;71(2):409–411. Reference Source

[ref-8] Denton A Yatsenko H Jay J : Sanger Tree of Life wet laboratory protocol collection V.1. *protocols.io.* 2023. 10.17504/protocols.io.8epv5xxy6g1b/v1

[ref-9] Di Tommaso P Chatzou M Floden EW : Nextflow enables reproducible computational workflows. *Nat Biotechnol.* 2017;35(4):316–319. 10.1038/nbt.3820 28398311

[ref-10] do Amaral RJV Bates A Denton A : Sanger Tree of Life RNA extraction: automated MagMax ^™^ mirVana. *protocols.io.* 2023. 10.17504/protocols.io.6qpvr36n3vmk/v1

[ref-11] Fell JW Guého-Kellermann E : *Guehomyces* Fell & Scorzetti (2004).In: *The Yeasts*.2011;1773–1775. 10.1016/B978-0-444-52149-1.00143-9

[ref-12] Fell JW Scorzetti G : Reassignment of the basidiomycetous yeasts *Trichosporon pullulans* to *Guehomyces pullulans* gen. nov., comb. nov. and *Hyalodendron lignicola* to *Trichosporon lignicola* comb. nov. *Int J Syst Evol Microbiol.* 2004;54(Pt 3):995–998. 10.1099/ijs.0.03017-0 15143054

[ref-13] GBIF: GBIF occurrence download - Tausonia pullulans. *GBIF Occurrence Download.* (August 31,2023); [Accessed 31 August 2023]. 10.15468/dl.pfxks2

[ref-14] Guan D McCarthy SA Wood J : Identifying and removing haplotypic duplication in primary genome assemblies. *Bioinformatics.* 2020;36(9):2896–2898. 10.1093/bioinformatics/btaa025 31971576 PMC7203741

[ref-15] Harry E : PretextView (Paired REad TEXTure Viewer): a desktop application for viewing pretext contact maps. 2022. Reference Source

[ref-16] Howe K Chow W Collins J : Significantly improving the quality of genome assemblies through curation. *GigaScience.* 2021;10(1): giaa153. 10.1093/gigascience/giaa153 33420778 PMC7794651

[ref-17] Jay J Yatsenko H Narváez-Gómez JP : Sanger Tree of Life sample preparation: triage and dissection. *protocols.io.* 2023. 10.17504/protocols.io.x54v9prmqg3e/v1

[ref-18] Kerpedjiev P Abdennur N Lekschas F : HiGlass: web-based visual exploration and analysis of genome interaction maps. *Genome Biol.* 2018;19(1): 125. 10.1186/s13059-018-1486-1 30143029 PMC6109259

[ref-19] Kim MJ Lee HW Kim JY : Impact of fermentation conditions on the diversity of White Colony-Forming Yeast and analysis of metabolite changes by White Colony-Forming Yeast in Kimchi. *Food Res Int.* 2020;136: 109315. 10.1016/j.foodres.2020.109315 32846523

[ref-20] Lindner P : Mikroskopische Betriebskontrolle in den Gärungsgewerben.Open Library,1901. Reference Source

[ref-21] Liu XZ Wang QM Göker M : Towards an integrated phylogenetic classification of the Tremellomycetes. *Stud Mycol.* 2015;81:85–147. 10.1016/j.simyco.2015.12.001 26955199 PMC4777781

[ref-22] Manni M Berkeley MR Seppey M : BUSCO update: novel and streamlined workflows along with broader and deeper phylogenetic coverage for scoring of eukaryotic, prokaryotic, and viral genomes. *Mol Biol Evol.* 2021;38(10):4647–4654. 10.1093/molbev/msab199 34320186 PMC8476166

[ref-23] Moylett EH Chinen J Shearer WT : Trichosporon pullulans infection in 2 patients with chronic granulomatous disease: an emerging pathogen and review of the literature. *J Allergy Clin Immunol.* 2003;111(6):1370–1374. 10.1067/mai.2003.1522 12789241

[ref-24] Nakagawa T Ikehata R Uchino M : Cold-active acid beta-galactosidase activity of isolated psychrophilic-basidiomycetous yeast *Guehomyces pullulans*. *Microbiol Res.* 2006;161(1):75–79. 10.1016/j.micres.2005.07.003 16338594

[ref-25] Narváez-Gómez JP Mbye H Oatley G : Sanger Tree of Life sample homogenisation: covaris cryoPREP ^®^ automated dry Pulverizer V.1 . *protocols.io.* 2023. 10.17504/protocols.io.eq2lyjp5qlx9/v1

[ref-26] Rao SSP Huntley MH Durand NC : A 3D map of the human genome at kilobase resolution reveals principles of chromatin looping. *Cell.* 2014;159(7):1665–1680. 10.1016/j.cell.2014.11.021 25497547 PMC5635824

[ref-27] Rhie A McCarthy SA Fedrigo O : Towards complete and error-free genome assemblies of all vertebrate species. *Nature.* 2021;592(7856):737–746. 10.1038/s41586-021-03451-0 33911273 PMC8081667

[ref-28] Rhie A Walenz BP Koren S : Merqury: reference-free quality, completeness, and phasing assessment for genome assemblies. *Genome Biol.* 2020;21(1): 245. 10.1186/s13059-020-02134-9 32928274 PMC7488777

[ref-29] Romero MC Hammer E Cazau MC : Isolation and characterization of biarylic structure-degrading yeasts: hydroxylation potential of dibenzofuran. *Environ Pollut.* 2002;118(3):379–382. 10.1016/s0269-7491(01)00290-1 12009135

[ref-30] Sampaio JP : *Tausonia* Bab’eva (1998).In: *The Yeasts*.2011;1999–2001. 10.1016/B978-0-444-52149-1.00159-2

[ref-31] Sláviková E Košíková B Mikulášova M : Biotransformation of waste lignin products by the soil-inhabiting yeast *Trichosporon pullulans*. *Can J Microbiol.* 2002;48(3):200–203. 10.1139/w02-013 11989763

[ref-32] Strickland M Cornwell C Howard C : Sanger Tree of Life fragmented DNA clean up: manual SPRI. *protocols.io.* 2023. 10.17504/protocols.io.kxygx3y1dg8j/v1

[ref-33] Surana P Muffato M Qi G : Sanger-tol/readmapping: sanger-tol/readmapping v1.1.0 - Hebridean Black (1.1.0). *Zenodo.* 2023a. 10.5281/zenodo.7755669

[ref-34] Surana P Muffato M Sadasivan Baby C : sanger-tol/genomenote (v1.0.dev). *Zenodo.* 2023b. 10.5281/zenodo.6785935

[ref-35] Trochine A Bellora N Nizovoy P : Genomic and proteomic analysis of *Tausonia pullulans* reveals a key role for a GH15 glucoamylase in starch hydrolysis. *Appl Microbiol Biotechnol.* 2022a;106(12):4655–4667. 10.1007/s00253-022-12025-7 35713658

[ref-37] Trochine A Bellora N Nizovoy P : Genomic and proteomic analysis of *Tausonia pullulans* reveals a key role for a GH15 glucoamylase in starch hydrolysis. *Appl Microbiol Biotechnol.* 2022b;106(12):4655–4667. 10.1007/s00253-022-12025-7 35713658

[ref-38] Todorovic M Oatley G Howard C : Sanger Tree of Life HMW DNA Extraction: Automated Plant MagAttract v.2, *protocols.io* .2023a. 10.17504/protocols.io.36wgq3n13lk5/v1

[ref-36] Todorovic M Sampaio F Howard C : Sanger Tree of Life HMW DNA fragmentation: diagenode Megaruptor ^®^3 for PacBio HiFi. *protocols.io.* 2023b. 10.17504/protocols.io.8epv5x2zjg1b/v1

[ref-39] Uliano-Silva M Ferreira JGRN Krasheninnikova K : MitoHiFi: a python pipeline for mitochondrial genome assembly from PacBio high fidelity reads. *BMC Bioinformatics.* 2023;24(1): 288. 10.1186/s12859-023-05385-y 37464285 PMC10354987

[ref-40] Vasimuddin M Misra S Li H : Efficient architecture-aware acceleration of BWA-MEM for multicore systems.In: *2019 IEEE International Parallel and Distributed Processing Symposium (IPDPS).*IEEE,2019;314–324. 10.1109/IPDPS.2019.00041

[ref-41] Yao H Tian S Wang Y : Sodium bicarbonate enhances biocontrol efficacy of yeasts on fungal spoilage of pears. *Int J Food Microbiol.* 2004;93(3):297–304. 10.1016/j.ijfoodmicro.2003.11.011 15163586

[ref-42] Yurkov AM : Yeasts of the soil - obscure but precious. *Yeast.* 2018;35(5):369–378. 10.1002/yea.3310 29365211 PMC5969094

[ref-43] Zhou C McCarthy SA Durbin R : YaHS: yet another Hi-C scaffolding tool. *Bioinformatics.* 2023;39(1): btac808. 10.1093/bioinformatics/btac808 36525368 PMC9848053

