# Supplementary material for: The genome sequence of a basidiomycete yeast, Tausonia pullulans (Lindner) X.Z. Liu, F.Y. Bai, M. Groenew. & Boekhout, 2016 (Mrakiaceae)
Source: Wellcome Open Res. 2024 Nov 20;9:685. [Version 1] doi: 10.12688/wellcomeopenres.23350.1 (PMC11803393; doi:10.12688/wellcomeopenres.23350.1)
Supplement: Peer review history

# Reviewer report: Reviewer response for version 1

**DOI:** 10.21956/wellcomeopenres.25735.r114597

**Published:** 2024-12-31

**Authors:** David Diaz Escandon

**Affiliations:** 1 University of Alberta, Edmonton, Alberta, Canada

The paper presents a—chromosome-level—genome release for the basidiomycete yeast, *Tausonia pullulans*. The authors properly explained  the rationale behind studying this organism, particularly given its recent reclassification to Cystofilobasidiales, and its nearly cosmopolitan distribution. The paper underscores the value of expanding our foundational knowledge of this yeast with this newly released genome, especially its enriched enzymatic machinery previously described in the literature and already applied to various industrial processes. The authors present a 23 Mb chromosome-level genome assembly derived from a single culture, utilizing state-of-the-art DNA extraction, sequencing, and assembly techniques, together with an 18 Kb mitochondrial genome. This represents the first and only chromosome-level assembly for this species; however, it is worth noting that four other genomes are available on NCBI.

 High-quality reference genome releases are highly demanded in the current era of rapidly advancing sequencing technologies. Chromosome-level assemblies have become the gold standard, providing a necessary framework for revising and corroborating scaffold-based assemblies. This premise alone adds significant merit to the paper. However, there are a few critical issues that need to be addressed:

                   1. **Lack of genome/species-specific analyses in the genome release**: While this is a genome release, its current state does not differ significantly from the information currently available on NCBI or EBI. Although additional information is provided in the methods section, many of the protocols referenced are for bulk sequencing/analysis and published in ‘protocols.io’ and Nextflow. The lack of genome-specific discussion or analysis is a notable limitation. This could be remedied by including comparative analyses with the other available genomes or by performing additional steps, such as genome annotation. Personalized genome releases should include unique insights or findings specific to the genome in question. This might seem trivial, but at this stage it seems like a personalized genome release of a bulk generated genome.

                   2. **Low BUSCO Completeness**: The reported BUSCO completeness of 29.9% (benchmark >95%) using TremellomycetesODB10 requires further discussion. While the paper asserts completeness in various ways, the current standard for genome completeness is based on BUSCO metrics, and a value of 29.9% needs to be either justified or explained. Completeness scores at the class level are often lower due to under-sampling in some classes or order selection biassed, but a value below 50% raises questions about either the genome’s completeness or its taxonomic assignment unless additional context is provided. That said, It is worth noting that the TremellomycetesODB10 dataset primarily represents *Cryptococcus* and includes only 18 reference genomes, most of them from Tremellales. This narrow representation suggests that any major evolutionary shifts between orders may not be well captured. To strengthen the paper, I recommend including an assessment using BasidiomycotaODB10, which should ideally score close to 100% (92% with Busco 5.8.2 and BasidiomycotaODB10), or incorporating a secondary completeness metric such as EukCC. It would be worth discussing this BUSCO results, regards of the other metrics included, since TremellomyctesODB10 includes *Trichosporon *(Trichosporonales), *Tausonia* former classification.  Additionally BUSCO completeness using version 5.8.2 and the latest updated of Tremellomycetes shows a higher completeness with 46.6%.

                   3. **Unutilized RNA Sequencing Data**: The inclusion of RNA extraction and sequencing methods in the paper, without corresponding results or analysis, is problematic. If this is intended as a personalized genome release, the paper would benefit greatly from leveraging this data. As a rule of thumb, data mentioned in the methods should be used in the results. In this case, the RNA sequencing data is mentioned only in “Data Availability”, with the suggestion that it will be used for future annotations. It would be more appropriate to either complete the annotations and include them in this genome release or exclude the RNA sequencing procedure entirely from the current manuscript.

Are sufficient details of methods and materials provided to allow replication by others?

Partly

Is the rationale for creating the dataset(s) clearly described?

Yes

Are the datasets clearly presented in a useable and accessible format?

Yes

Are the protocols appropriate and is the work technically sound?

Yes

Reviewer Expertise:

Lichens, Genomics, Evolution

I confirm that I have read this submission and believe that I have an appropriate level of expertise to confirm that it is of an acceptable scientific standard, however I have significant reservations, as outlined above.

# Reviewer report: Reviewer response for version 1

**DOI:** 10.21956/wellcomeopenres.25735.r114599

**Published:** 2024-12-26

**Authors:** Reyim Mamut

**Affiliations:** 1 Xinjiang University, Urumqi, Xinjiang, China

In terms of the significance of this article, it is delivers an important contribution to the genome assembly of *Tausonia pullulans* . Basidiomycete yeast have wide application value in industrial and agricultural production, biopharmaceuticals, and environmental protection. For example, they play an important systematic role in fungal evolution and have significant potential applications in developing low-temperature industrial enzymes and constructing low-temperature exogenous protein expression systems. Therefore, studying its genome is a necessary task.

 The author provided a detailed Genome sequence report, outlining the standard process for obtaining genomes of *Tausonia pullulans* *.* *T* *he methods:* Sample acquisition and nucleic acid extraction,Sequencing,Genome assembly, used in the article are reasonable and reflect a high level of genomic research.  

 Recently, the number of species of basidiomycetes yeast has been increasing, and some new groups with special morphologies have been discovered, which have filled many gaps in the phylogenetic tree.

 Therefore, in my opinion, the section on phylogenetic analyses is needed. Phylogenetic analyses could clarify the evolutionary relationships of *T. pullulans* within the Tremellomycetes. The improvement of this part of the content will help to better understand of *T. pullulans* . On the other hand, it will reflect the value of genomic research.

Are sufficient details of methods and materials provided to allow replication by others?

Yes

Is the rationale for creating the dataset(s) clearly described?

Yes

Are the datasets clearly presented in a useable and accessible format?

Yes

Are the protocols appropriate and is the work technically sound?

Partly

Reviewer Expertise:

Taxonomy, Lichenology and Bioinformatic

I confirm that I have read this submission and believe that I have an appropriate level of expertise to confirm that it is of an acceptable scientific standard.

# Reviewer report: Reviewer response for version 1

**DOI:** 10.21956/wellcomeopenres.25735.r113616

**Published:** 2024-12-10

**Authors:** David Pizarro

**Affiliations:** 1 Universidad Complutense de Madrid, Madrid, Community of Madrid, Spain

The article delivers an important contribution by presenting the genome assembly of *Tausonia pullulans*, a yeast species with notable ecological and industrial relevance. The authors have successfully assembled a genome spanning 23.9 Mb, which includes 20 chromosomal pseudomolecules and a complete mitochondrial genome. Employing cutting-edge sequencing techniques like PacBio HiFi reads and Hi-C scaffolding, combined with manual curation, highlights the thoroughness of the work. Additionally, integrating the genome within the broader context of the yeast’s enzymatic and ecological capabilities provides meaningful insights, especially for potential biotechnological applications such as lignocellulose degradation and pollutant processing. The availability of raw data and genome assembly files in public repositories also reinforces the accessibility and reproducibility of the study, which are significant strengths.

 Despite these contributions, the study faces a critical challenge in the form of its low genome completeness, as reflected by a BUSCO score of 29.9%. This figure, which is well below the typical standard for reference genomes, raises questions about the representativeness of the assembly. Missing conserved single-copy orthologs may indicate technical challenges, such as an inability to resolve complex or repetitive genomic regions, or errors during assembly that led to the collapse of haplotypes. Alternatively, *T. pullulans* may exhibit biological genome reduction, as seen in certain other fungal species. If the latter is the case, presenting supporting evidence for such reduction would greatly enhance the study’s impact. To address these issues, a reanalysis of the data using alternative pipelines, such as tools that are better suited for resolving heterozygosity or repetitive sequences, could improve genome completeness. Incorporating additional sequencing technologies, like ultra-long reads, may also help resolve ambiguities and gaps.

 The functional annotation of the genome could be further developed to strengthen the study’s claims about enzymatic activities and their biotechnological potential. While the manuscript references the yeast’s role in processes like starch hydrolysis and lignocellulose degradation, these assertions are primarily based on previous research rather than novel findings derived directly from the genome. Enhanced annotation using tools such as InterProScan or KEGG would allow the authors to identify specific genes or pathways tied to these activities, offering new insights into the functional capabilities of *T. pullulans*. Furthermore, improving the annotation could help clarify whether the low BUSCO score is due to assembly gaps or a lack of functional gene content.

 Another area for improvement is the evolutionary context provided in the study. While the authors offer a comprehensive account of the species’ taxonomic history, the manuscript could be significantly enriched by using the assembled genome for phylogenetic analyses. Such analyses could clarify the evolutionary relationships of *T. pullulans* within the Tremellomycetes and identify traits or adaptations that distinguish it from closely related species. Insights into genome architecture, potential signs of metabolic specialization, or evidence of genome reduction could make the study more impactful.

 Moreover, the data visualization and interpretation within the manuscript require greater attention. Figures such as BlobToolKit plots and Hi-C maps are useful tools for assessing genome assembly quality but are not sufficiently analyzed in the current text. Offering more detailed explanations of these visualizations and linking them to broader conclusions about the genome’s structure and organization would help readers better understand the study’s findings. Expanding on the mitochondrial genome analysis could also reveal interesting aspects of the species’ biology or adaptations.

 In conclusion, while the genome assembly of *T. pullulans* presented in this article is a valuable resource, addressing the low BUSCO score, improving functional annotation, integrating phylogenetic analyses, and enhancing data interpretation are essential steps. These revisions would elevate the study, ensuring it becomes a robust and widely useful reference for fungal genomics and biotechnology. By tackling these challenges, the article can fulfill its potential to significantly advance research in these fields.

Are sufficient details of methods and materials provided to allow replication by others?

Yes

Is the rationale for creating the dataset(s) clearly described?

Yes

Are the datasets clearly presented in a useable and accessible format?

Yes

Are the protocols appropriate and is the work technically sound?

Partly

Reviewer Expertise:

Genomic, Lichenology and Bioinformatic

I confirm that I have read this submission and believe that I have an appropriate level of expertise to confirm that it is of an acceptable scientific standard, however I have significant reservations, as outlined above.
